# Maternal-infant antibiotic resistance genes transference: what do we know?

**DOI:** 10.1080/19490976.2023.2194797

**Published:** 2023-04-05

**Authors:** Anna Samarra, Maria Esteban-Torres, Raul Cabrera-Rubio, Manuel Bernabeu, Silvia Arboleya, Miguel Gueimonde, Maria Carmen Collado

**Affiliations:** a Department of Biotechnology, Institute of Agrochemistry and Food Technology- National Research Council (IATA-CSIC), Valencia, Spain; b Vicerectorat de Recerca, Universitat de Barcelona (UB), Barcelona, Spain; c Department of Microbiology and Biochemistry, Dairy Research Institute- National Research Council (IPLA-CSIC), Villaviciosa, Spain

**Keywords:** Antibiotic, resistome, microbiota, gut, breastmilk, infant, mother, pregnancy

## Abstract

Resistance to antibiotics is becoming a worldwide threat as infections caused by multidrug-resistant pathogenic microorganisms can overcome antibiotic treatments and spread quickly in the population. In the context of early life, newborns are at increased risk as their immune system is still under development, so infections and acquisition of resistance during childhood have short- and long-term consequences for the health. The moment of birth is the first exposure of infants to possible antibiotic-resistant microorganisms that may colonize their gut and other body sites. Different factors including mode of delivery, previous antibiotic exposure of the mother, gestational age and consumption of antibiotics in early-life have been described to modulate the neonate’s microbiota, and thus, the resistome. Other factors, such as lactation, also impact the establishment and development of gut microbiota, but little is known about the role of breastmilk in transferring Antibiotic Resistant Genes (ARG). A deeper understanding of vertical transmission of antibiotic resistance from mothers to their offspring is necessary to determine the most effective strategies for reducing antibiotic resistance in the early life. In this review, we aim to present the current perspective on antibiotic resistances in mother-infant dyads, as well as a new insight on the study of the human gut and breastmilk resistome, and current strategies to overcome this public health problem, toward highlighting the gaps of knowledge that still need to be closed.

## 1. Antibiotics in mother-infant dyads

Antibiotic resistance occurs when microorganisms resist the effects of antibiotics, making them no longer effective in treating bacterial infections. This occurs as bacteria evolve and develop new strategies to counteract the impact of antibiotics, leading to the rise of Antibiotic-Resistant Microorganisms (ARMs)^1^. Resistance to antibiotics is a natural evolutionary process for microorganisms, but its progression is accelerated by the selective pressure exerted by the excessive use of antibacterial drugs^2^. Incorrect prescription, over-consumption and prolonged use of antibiotics in humans and animals have affected the microbiota equilibrium, resulting in an increased risk of encountering health issues in the future, and caused a global crisis of antibiotic resistance^3^. Indeed, the creation of a new antibacterial drug is soon followed by the emergence of a resistance to it. ARMs are widespread and can be easily transmitted from person to person or from animals to humans, including through consumption of animal-derived food^4^. Antibiotic-resistant bacteria carry a set of Antibiotic Resistance Genes (ARGs), thus allowing the bacteria to overcome the antibiotic effects. The set of ARGs present in a population of bacteria is known as the resistome.

The World Health Organization (WHO) has declared that the rise of antibiotic resistances around the world, including high- and low-income countries, is a major threat. To address this challenge, a comprehensive global action plan has been put in place to ensure that prevention and treatment of infectious diseases are carried out using safe and effective medicines^5^. Antibiotics are becoming increasingly ineffective due to the fast spread of drug resistances globally. As a result, hospital stays become extended and infected patients require more expensive and intensive care. The increasing prevalence of antibiotic-resistant microorganisms complicates medical procedures such as surgeries, chemotherapy, and organ transplants^5^. Accordingly, the WHO and the US Centers for Disease Control and Prevention (CDC) classify ARMs as a threat to human health worldwide^6^. Despite its importance, ARM infections are currently underreported, but it is estimated that there are more than 2.8 million AMR infections and up to 35,000 deaths per year in the United States alone, with an estimated derived cost of more than $4.7 billion^7^. In Europe, more than 33,000 deaths are attributed to acquired ARM infections each year, representing $1.5 billion in direct and indirect costs^8^. Furthermore, in developing countries, communicable diseases continue to be a leading cause of death, with a rise in newly emerging and reemerging infections^9^.

In 2017, the WHO published a list of 12 bacterial families for which new antimicrobial development is urgently needed^10^. Bacteria were classified into three categories based on the urgency to develop novel antibiotics to combat them: critical, high and medium priority^11^. The pathogens included in the most critical group are multidrug-resistant bacteria that are threat to hospitalized patients, causing nosocomial infections^12,13^. *Enterococcus faecium, Staphylococcus aureus, Klebsiella pneumoniae, Acinetobacter baumannii, Pseudomonas aeruginosa*, and *Enterobacter* species, describing the ESKAPE group pathogens, are recognized as a top priority for treatment^14^.

These pathogens are incredibly difficult to treat with antibiotics and can lead to severe, life-threatening illnesses such as blood infections and pneumonia^15^. Among these pathogens, extended-spectrum β-lactamase (ESBL) and carbapenem-resistant *P. aeruginosa, A. baumannii*, *K. pneumoniae* and *Enterobacter spp*., are considered critical priority pathogens. On the other hand, the high priority group includes vancomycin- and methicillin-resistant *S. aureus* (MRSA and VRSA) and vancomycin-resistant *E. faecium* (VRE)^10^.

Early human life (the perinatal and postnatal period) represents the first exposition to antibiotics, and at same time, it is when microbiota establishment and development take place in parallel with other biological processes (immune maturation, metabolic and neurocognitive processes). It is estimated that in high-income countries, 70% of all infants receive at least one course of antibiotics during the first year of life to treat skin, respiratory and gastrointestinal infections^16^. In low and middle-income nations, the use of antibiotics is higher, with children estimated to take an average of 11 rounds of antibiotics within their first 2 years of life^17^. Furthermore, the 80% of the drugs a women is subjected during pregnancy are antibiotics, and 35% of pregnant women in Western countries are exposed to antibiotics^18^. Pregnant mothers can receive antibiotics during the latter stages of pregnancy to prevent bacterial infections in their genital tract which could harm both the mother and the child^19^. Many women are exposed to antibiotics during delivery with the prophylaxis to prevent the vertical transmission of group B streptococci being the main reason for intrapartum antibiotics administration. For women conducting cesarean section procedures, the WHO recommends using a single dose of first-generation cephalosporin or penicillin^20^. For those with uncomplicated vaginal birth and without any associated risk factor routine antibiotic prophylaxis is not recommended^21^. Table 1 summarizes the most commonly used antibiotics during pregnancy and lactation and their indication of use.

**Table 1.** Antibiotics commonly used during the perinatal period.

Antibiotic	Class of antibiotic	Target infection
Penicillins^22^	β-lactams	Group B streptococci^22^ infections, respiratory infections, urinary tract and skin infections, septicemia.
Cephalosporin^23^		Respiratory tract, urinary tract and skin infections, gonorrhea.
Carbapenems^24^		Gram-negative bacterial infections of the urinary tract, kidney, gastrointestinal tract, respiratory tract, and urogenital and pelvic area.
Azithromycin, erythromycin^25^	Macrolides	Respiratory, intestine, gynecological, urogenital and skin infections, chlamydia infections and syphilis.
Ciprofloxacin, norfloxacin^26,27^	Quinolones	Serious gram-negative bacterial infections.
Clindamycin^28,29^	Lincomycin	Respiratory, skin, blood, female reproductive organ, and internal organ infections.
Gentamycin, streptomycin^30,31^	Aminoglycosides	Pyelonephritis, urinary tract infection, gram-negative bacterial infections.
Metronidazole^32^	Nitroimidazole	Infections affecting the reproductive system, gut, skin, heart, bones, joints, lungs, blood, and nervous system.
Vancomycin^33,34^	Glycopeptide	Colitis, *C. difficile*–induced diarrhea, gram-positive bacterial infections.
Trimethoprim^35^	Dyaminopyrimidines	Urinary tract infections, pneumonia, diarrhea.

The risk of spreading antibiotic-resistant pathogens is particularly severe in newborns as their gut microbiota and immune system are not as mature and robust as those of adults. The worldwide problem of antibiotic resistance results in approximately 214,000 yearly newborn fatalities from septic infections, highlighting its devastating impact on infant health^36^.

Thus, the use of antibiotics in early stages of life can influence the microbiota-host crosstalk, resulting in a dysbiosis that can lead to short-term and long-term consequences^37,38^. Reported short-term consequences in premature infants include a correlation between decreased diversity of microbiota following an antibiotic treatment with and increased frequency of sepsis^39^. In addition, early antibiotic treatment in premature babies with extremely low body weight has been linked with an increased risk of necrotizing enterocolitis and death^40–42^.

Long-term consequences of early antibiotic exposure include pathologies related to the immune system maturation, and disruptions in the metabolic functions of the microbiota. Immune disorders, such as allergies, asthma, eczema or celiac disease, have been reported as a consequence of pre- and postnatal exposure to antibiotics^43–47^. Research has also established connections between early and repeated exposure to antibiotics and growth^48^, risk of obesity^49–51^ and inflammatory bowel disease^52^.

## 2. The maternal-infant resistome

The gut microbiome of adult humans is a source of ARGs that opportunistic pathogens can acquire. Bacterial pathogens carrying ARGs in pregnant mothers may impact ovum implantation, the pregnancy suspension, and delivery. This also increases the chance of early infections in the newborn and affects the establishment and development of the infant’s gut microbiome^53^.

The composition of the gut microbiota is a key factor in determining the presence of ARGs, as most of these genes are carried by a few specific taxa. Studies employing culture-based or metagenomics methods have shown that Gammaproteobacteria in the Pseudomonadota phylum are major carriers of ARGs, while Bacillota is also enriched in pregnant women and can carry ARGs^53,54^. Correlations between the phylum Proteobacteria and ARGs/mobile genetic elements (MGEs) have been found^55,56^. In addition, Proteobacteria are capable of DNA transfer through conjugation, which plays a critical role in the spread of ARGs^57^. The most abundant and unique ARGs in the infant gut belong to *Escherichia, Klebsiella, Citrobacter*, and *Enterobacter* species^58^.

On the contrary, *Bifidobacterium* spp. are negatively correlated with antibiotic resistance load^15^. Members of this genus are relevant commensals that dominate the breastfed infant gut microbiome, primarily due to their ability to use human milk oligosaccharides (HMOs) and other carbohydrates efficiently^59,60^. *Bifidobacterium* species are less likely to possess ARGs than other taxa such us *Streptococcus, Staphylococcus, Enterococcus* and *Bacteroides* spp.^61,62^.

Metagenomic shotgun sequencing has revealed the high levels of ARGs in the gut microbiome of infants compared to adults, even when not exposed to antimicrobials^15,56,63–65^. This is likely due to the main role of Pseudomonadota in the infant microbiota during the first months of life, being later replaced by other bacterial phyla. Further, infant resistome analyses at different time points have elucidated higher relative abundances of ARGs in younger infants in comparison to older children^66,67^. Different factors including delivery mode, type of breastfeeding and antibiotic exposure influence the ARG load in infants. Thus, the development of the infant’s resistome is linked to the evolution of the microbial composition, which contributes to the dynamics and stabilization of the infant resistome to adulthood^67,68^.

In this regard, the human resistome also includes the mobilome, which is defined as all Mobile Genetic Elements (MGEs) in a cell which are able to move around within or between genomes of different organisms^68^. They include plasmids, integrons, bacteriophages and transposable elements^69^.

There is evidence that an increased presence of MGEs in infant feces may facilitate the transmission of ARGs among and between species^15^. In the context of mother-infant transfer, it has been seen that the acquisition of the mobilome in early stages of life may be due to maternal influence and/or family-specific environment, with the latter being the most important influence at 6 months of life^70^.

The increasing use antibiotics during pregnancy and breastfeeding constitutes a risk factor for the development of ARM in the mother’s resistome, which can then be passed on to the offspring and impact their resistome^71^. Although load and variety of antibiotic resistance may vary between groups of infants according to different early-life factors, most predominant ARGs confer resistance to beta-lactams, tetracyclines, macrolides, aminoglycosides and quinolones^72^.

### Beta-lactamases

Beta-lactamases have been prescribed for many decades, and are commonly used during pregnancy and labor, thus it is predictable that resistance appears in infant fecal samples^58,72^. The majority of beta-lactamases genes are extended-spectrum beta-lactamases (ESBLs), that can be grouped in: TEM beta-lactamases (class A), OXA beta-lactamases (class D), SHV beta-lactamases (class A) and CTX-M beta-lactamases (class A)^73^. ESBL-producing Enterobacteriaceae (ESBL-E), including *Escherichia coli (E. coli)* and *Klebsiella pneumoniae*, are a public health concern as they can potentially contribute to the neonatal mortality, and they are ranked as a priority among pathogens in need of new treatment by the WHO^74^. These multidrug-resistant bacteria cause a mortality rate of more than 70% in infants as they are widely spread in the gut microbiome^75^.

### Tetracyclines

Genes conferring resistance to tetracycline (*tet* genes) have been reported to be the most abundant among infant fecal samples and are therefore the most representative resistant genes in the infant resistome^76^. The *tet* genes are the most frequently shared ARGs between mothers and infants, indicating that they are easily transmitted from the mother to the newborn^56,58,71,77^. The prevalence of tetracycline-resistant genes in various bacteria species, coupled with the common utilization of tetracycline to treat infections, may explain the acquisition of *tet* genes by women. Although this antibiotic is not recommended during pregnancy and early life due to potential dental staining effects, it is still extensively used in animals and, therefore, the environment and the diet may be sources of *tet* genes without direct exposure to the antibiotic^77^.

### Macrolides

Macrolides are commonly used against infections in infants, such as trachoma, pertussis and *Campylobacter* enteritis^49,78^. Clindamycin and erythromycin are used to treat infections in skin and tissues produced by Staphylococci. Nevertheless, the expression of macrolide-lincosamide-streptogramin B resistance (MLSB) can reduce the efficacy of these antibiotics. The accumulation of macrolide resistance genes in the gut microbiome of newborns could present future consequences for their health. Klassert *et. al* ^72^ found that genes for macrolide resistance (*mefA, ermB*, and *ermC*) are the most widely distributed among mother-infant pairs and have the greatest likelihood of being transmitted vertically^79^.

### Aminoglycosides

Aminoglycosides, usually combined with a beta-lactam, are widely used for neonatal sepsis. Antibiotics such as gentamicin, streptomycin, kanamycin, neomycin are examples of aminoglycosides. It has been identified in the infant gut microbiota the presence of genes for acetylation, phosphorylation and adenylation conferring aminoglycoside resistance^80^. The *aac(6′)-aph(2′′), aadE* and *aph(3′)-I* genes are the most commonly detected ARGs against aminoglycosides during the first year of life^73^.

### Quinolones

Quinolone antibiotics are synthetic and widely used in medicine. Like other antibiotics, resistance to quinolones has emerged due to their frequent use, and it is now common in some bacterial pathogens. Although quinolones are rarely used in pediatrics, the presence of ciprofloxacin-resistant *E. coli* has been found in the feces of infants^81^. Additionally, when mothers are prescribed quinolones, there is an increased risk of their offspring acquiring community-acquired, quinolone-resistant *E. coli* ^82^.

## 3. Antibiotic resistance acquisition and transmission in early stages of life

**Figure 1.**
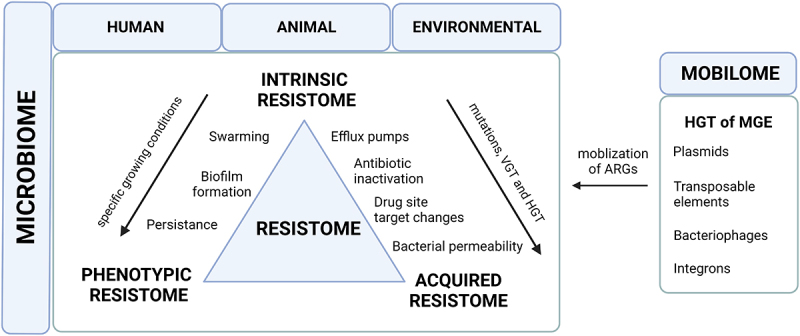
A schematic overview of the human resistome and mobilome. Intrinsic resistance is a property controlled by chromosomes and is related to the general physiology of the microorganism. The acquired resistance is encoded on plasmids and may be classified into four mechanisms: changes in the cell wall that make it less permeable to antibiotics, modifications of enzymes that inactivate antibiotics, changes in the target site of the drug, and efflux pumps that remove antibiotics from the cell. Phenotypic bacterial resistance appears in three categories: persistence, where a subpopulation of bacteria survive even though the majority is inhibited by the antibiotic; formation of biofilms, where bacteria form communities protected by a matrix; and swarming, where cells become hyper-flagellated, allowing them to colonize nutrient-rich environments and become less susceptible to antibiotics. MGE: mobile genetic elements; HGT: horizontal gene transfer; VGT: vertical gene transfer; ARG: antibiotic genetic elements. .

It is important to distinguish between intrinsic, acquired and adaptative antibiotic resistance to determine how the resistance develops (Figure 1). Intrinsic resistance is naturally present in the microorganism; it is chromosomically encoded and is connected to the microorganism physiology^83^. The intrinsic resistance is conferred by several elements that contribute to antibiotic resistance, independent of earlier exposure to antibiotics, and can only be vertically transmitted^84^. These genes may be captured by MGEs during evolution and become mobile^85^. The acquired resistance is based on mutations in the bacterial chromosomic DNA and on the acquisition of mobile resistance genes, by either horizontal or vertical gene transfer. Adaptative resistance refers to a transient situation in which a susceptible bacterial population becomes resistant to antibiotics^84^.

Despite their widespread presence in bacteria, not all bacteria harbor ARGs at comparable levels. In early stages of life, antibiotic resistance can be a result of horizontal transfer of genes between bacteria, vertical transmission from the mother during pregnancy, birth and lactation and/or antibiotic exposure of the infant^71^. The availability of new methods, especially culture-independent strategies, have enabled new insights on antibiotic resistance transmission (Box 1).

[Box 1. Methods to study the resistome] **- Culture-based approaches**. This methodology continues to be essential to determine resistance phenotypes to multiple antimicrobials^30^. This approach stands alone or can also be used as a complement to metagenomic work by overcoming the depth bias inherent in metagenomic sequencing^31^. This methodology is only applicable in cultivable bacteria, not allowing the discovery of distant or unknown elements.

**- Whole-genome sequencing**. This methodology has high sensitivity, detecting species with low abundance and identifying potential ARGs. However, the full complexity of the resistome cannot be observed in vivo. Thus, it is necessary to validate using culture-based approaches to reduce the risk of false positives and determine that they are genuine resistance genes.

**- Complete metagenomic sequencing**. Methodology with which you can observe all the ARGs that would exist in a certain environment, taking into account that could be oversampled, because when these genes are overexpressed in a heterologous host may not be genuine resistance genes in their native contexts^32^.

**- Long-read sequencing techniques**. Previous research has demonstrated that utilizing long-read sequencing techniques can provide insight into the presence of ARGsin the resistome. Using a combination of both long-read (Oxford Nanopore) and short-read (Illumina) sequencing methods has been found to be an effective way to thoroughly examine the resistome in both human samples^33^ and in bacterial isolates^34^.

**- Single-cellsequencing techniques.** Refers to the process of analyzing the genomic, transcriptomic, and metabolomic functions of an individual cell through sequencing its genome or transcriptome^35^. Despite its high cost, this technique has been demonstrated as a powerful toolfor investigating non-cultivable organisms^86^.

**- Databases**. The detection and analysis of genetic elements require ongoing improvement, as it is crucial to track the development of resistance and its evolution over time. To achieve this, resistome databases must be frequently updated to incorporate newly discovered variations, such as variant sequences, insertions, and deletions, thereby enhancing our knowledge of resistance variations^87^. The most frequently cited specialized databases for ARGs that allow input of metagenomic data are ResFinder^88^, MEGARes^89^ and Resfams^90^.

### Vertical transfer of antibiotic resistance from mother to offspring

The source of antibiotic-resistant bacteria in infants remains unclear, as they can be transmitted from other individuals, the mother or even the environment^15^. The resistance profiles of mothers and infants can differ in terms of prevalence and specific resistant strains, but there is evidence of vertical transmission of ARGs from mother to offspring. Many ARGs occur only in either maternal or in child samples^56^.

The resistome of infants, which is the collection of antibiotic resistance genes in the gut, was found to have resistance to several broad-spectrum beta-lactam antibiotics, such as aztreonam, piperacillin-tazobactam, and cefepime^91^, but ARGs to these antibiotics were not present in the mothers’ resistome. Despite the difference between the mothers’ and infants’ resistomes, there is evidence of vertical transmission of antibiotic resistance genes from mothers to infants^15,92^. In a cohort of 50 samples, 59 ARGs of a total of 249 had at least one significant family transmission^56^. Kozak *et al*.^93^ showed that highly similar isolates of antibiotic-resistant *Lactobacillus fermentum*, *Lactobacillus gasseri*, *Bifidobacterium longum* and *Enterococcus faecalis* were found in both mothers and infants, indicating that resistant strains were passed to infants through vertical transmission in early life.

#### Vertical transmission of ARGs at birth

ARGs have been found in neonates within hours of birth, indicating that colonization of the newborns with ARMs can occur at birth or soon after, likely through contact with the mother’s uterus and skin or from the hospital environment^94^. As reviewed by Patangia *et al*. ^71^, some studies have shown that ARMs can be present in a mother’s vagina and be transferred to her offspring. These include *E. coli* resistant to ampicillin and cefotaxime-resistant and multidrug-resistant *E. coli* ^95–97^, penicillin-resistant *Streptococcus* ^98^, and tetracycline-resistant bacteria including *Prevotella*, *Lactobacillus*, *Ureaplasma*, *Gardnerella, Corynebacterium* and *Staphylococcus* spp. carrying *tetM*, *tetO* and *tetW* genes^99^. However, there is still little evidence on the transmission of antibiotic resistance during pregnancy and birth and the associated health implications^71^.

#### Vertical transmission of ARGs during lactation

Breastfeeding provides numerous benefits for infants, including the transfer of maternal immune cells and antibodies, as well as vital nutrients and bacteria^100^. Human milk serves as a consistent source of commensal and potentially probiotic bacteria for the newborn’s gut, with *Staphylococcus* and *Streptococcus* spp. being the most dominant genera in a woman’s milk, followed by *Lactobacillus* and *Bifidobacterium* spp.^101^.

Human milk has been found to contain antibiotic-resistant bacteria. Studies have shown the presence of antibiotic-resistant strains of *Staphylococcus, Streptococcus, Acinetobacter, Enterococcus*, and *Corynebacterium* in human milk^15,102^. Multi-drug resistant profiles have also been detected in these species^100^.

Antibiotic resistant bacteria found in human milk have been linked to the administration of certain antibiotics to mothers during lactation, such as gentamicin, chloramphenicol, streptomycin, and quinupristin. Additionally, resistance to vancomycin, ampicillin, amoxicillin, oxacillin, and cephalothin has also been observed in human milk^100^. Multi-drug resistant strains of *Streptococcus* and *Staphylococcus* have been detected to be transferred to infants through breastfeeding^103–105^. This is concerning as *Staphylococcus aureus* is a major source of community and hospital infections^106^.

Metagenomic analyses have highlighted the high levels of ARGs and MGEs in breastmilk and the resemblance of the infant resistome to the mother’s milk resistome^15^. Antibiotic-resistant bacteria were observed in breastmilk as a potential source of ARM in infants [10]. Pärnänen *et al*.^15^ showed that infants shared gut resistomes and mobilomes with their own mother’s breastmilk microbiota with 70% of the ARGs detected in milk present in infant feces. Therefore, a mother can impact the infant’s gut resistome by transmitting resistant bacteria through her milk, although the resistance genes can also come from other parts of her body or external sources. The research found that most ARGs in the infant gut are carried by a limited number of microorganisms, and that antibiotics exposition has a major effect on the amount of resistance genes present during infancy.

Additional research is necessary to examine the transfer of antibiotic resistance from a mother to her infant, as it can affect the establishment of gut colonization. This will shed light into the source and dissemination of resistance genes and assist in the development of strategies to reduce this type of transmission.

## 4. Factors affecting antibiotic resistance in infants

**Figure 2.**
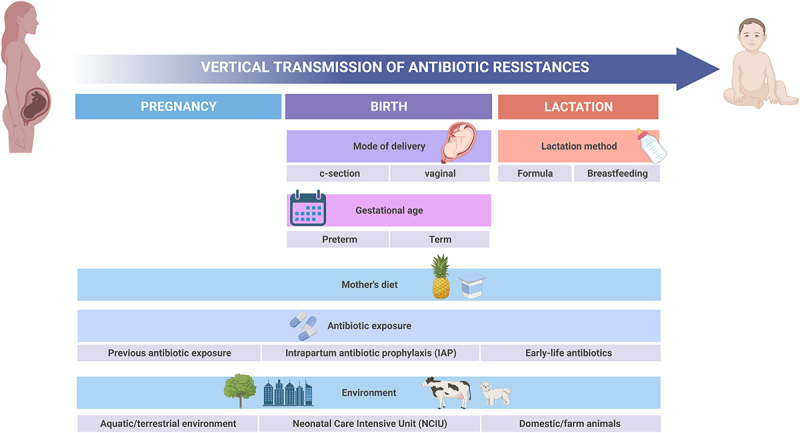
Factors influencing antibiotic resistance acquisition and vertical transmission.

Culture-based and metagenomic analyses have reported that the ARG load in the infant gut resistome can be affected and/or modulated by the type of feeding (maternal lactation or formula feeding), the duration of lactation, gestational and infant age (especially for preterm infants) and the antibiotic exposure in early stages of life (Figure 2).

### Gestational Age

Premature infants are of particular interest as they often undergo prolonged and early antibiotic treatment soon after birth, which can significantly alter the gut microbiome^107^.

The amount of ARGs in a preterm infants is influenced by their gestational age, with a tendency to have a higher ARG load^108^.

Premature infants carry a gut microbiota characterized by multidrug-resistant strains of *Klebsiella*, *Enterobacter*, and *Escherichia*, which are related with hospital infections and in neonatal intensive care units (NICUs)^63,109^. A study conducted by Rose *et al*. ^110^ found several ARGs in fecal samples from preterm neonates. The average number of ARGs per infant was 13 and the identified bacteria included *K. pneumoniae, C. perfringens*, and members of the *Enterobacter* and *Staphylococcus* genera. The presence of these genes in healthy premature infants emphasizes the importance of understanding the potential impact it may have on future medical treatment plans.

### Mode of Delivery

It is known that mode of delivery has an effect on neonatal microbiota development^111^. The gut microbiota of vaginal-delivered infants differs from those born via c-section over the first year of life, with higher levels of *Bifidobacterium* spp. and lower levels of *Klebsiella* and *Enterococcus* found in vaginally delivered infants^112,113^. Alterations in the composition of vaginal microbiota can have a substantial impact on the newborn’s gut microbiota^15^. Moreover, c-section delivery has been associated with reduced breastmilk microbiota diversity and richness^114–116^.

Recently, it has been shown that the way an infant is born and the amount of antibiotics they are exposed to during the perinatal period can affect the development of the gut microbiome establishment and have long-term impacts on their health, such as increasing the risk of asthma, inflammation, and obesity^117,118^.

Therefore, as delivery mode shapes maternal-infant and breastmilk microbiota, it can also influence the maternal, infant and milk resistome^119^. The WHO suggests that women who undergo a c-section should receive a single dose of penicillin or a first-generation cephalosporin as a preventive measure, instead of other types of antibiotics^120^. Some studies have elucidated the presence of ARGs in cesarean-section born babies^121^. Post-cesarean section periotonitis and surgical site infections have been related with higher rates of antibiotic resistance microorganisms in mothers^122,123^. However, there is no clear evidence on how mode of delivery may have an impact ARG load in breastmilk and on the mother-infant resistome development, and more research is needed to understand how mode of delivery, together with IAP, gestational age, maternal diet and other factors can shape the early-life resistance load.

### Early-life feeding-type

Breastfeeding provides numerous benefits for infants, including the transfer of maternal immune cells and antibodies, and it serves as a consistent source of commensal and potentially probiotic bacteria for the newborn’s gut, being the genus *Lactobacillus, Bifidobacterium*, *Staphylococcus* and *Streptococcus* the most dominant genera in a woman’s milk^101^. Nevertheless, breastmilk plays a crucial role on the acquisition of antibiotic resistances in early-life. Human milk has been found to contain species of *Staphylococcus, Streptococcus, Acinetobacter, Enterococcus*, and *Corynebacterium* resistant to antibiotics and with multidrug-resistant profiles have been isolated in human milk^15,100,102^.

On the other hand, infants who are exclusively breastfed have a more varied gut microbiome and fewer antibiotic-resistant bacteria, compared to those who are formula-fed. Formula feeding can cause changes in the gut environment leading to a simpler microbiome dominated by bacteria carrying antibiotic resistance genes^108^. A study that analyzed the gut metagenomes of 46 neonates found that compared to breastfeeding, formula feeding was associated with a 70% increase in the abundance of ARGs. Infants only fed with human milk had a more diverse gut microbiome and are less likely to be exposed to antibiotic-resistant bacteria, compared to infants who are fed formula. The study showed that formula feeding is associated with an increase in the presence of antibiotic-resistant genes and a decrease in the presence of bacteria that are typically found in a healthy gut microbiome. The families of obligate anaerobic bacteria, including *Veillonellaceae, Bifidobacteriaceae, Lachnospiraceae, Clostridiaceae*, and *Porphyromonadaceae*, were reduced in formula-fed infants. On the other hand, the populations of facultative anaerobic and aerotolerant bacteria, such as *Enterobacteriaceae, Enterococcaceae* and *Staphylococcaceae* increased in these infants. Formula-fed neonates had clinically significant ARGs that could potentially lead to methicillin resistance in *Staphylococcus aureus* and ESBL phenotypes, compared to those who were exclusively fed with human milk^108^.

In addition, duration of breastfeeding has been reported to influence the load of ARGs. Breastfeeding for less than 6 months has been correlated with an enrichment of ARGs and MGE. Breastfeeding for at least 6 months decreases the abundance of Gammaproteobacteria, and as a result, the abundance of ARGs, and conversely increases the abundance of *Bifidobacterium* spp.^15^.

### Antibiotic exposure during pregnancy, delivery and early life

Didier *et al*.^124^ reported that the use of antibiotics during the antenatal period can also increase the risk of antibiotic-resistant infections. In this regard, Li and collaborators reported that perinatal antibiotics impacted the transfer of bacteria from mothers to infants during the early stages of colonization. The administration of antibiotics led to an increase of horizontally transmitted bacteria from the environment, some of which were harmful strains commonly found in hospitals. In the group of mothers treated with antibiotics, the main source of ARGs in their offspring was not the microbiome of the maternal gut, while in the control group, most ARGs were passed on from the mother. This effect was particularly pronounced in the first 3–7 days after birth^125^.

Intrapartum Antimicrobial Prophylaxis (IAP) is a common clinical procedure during delivery that is used to prevent early-onset Group-B-*Streptococcus* (GBS) infections and in cases of pre-labor rupture of membranes or C-section deliveries. It is present in more than 30% of labors and is by far the most common exposure way to antibiotics in the perinatal and neonatal stages^126,127^. The effect of this treatment on the gut microbiome has gained attention the last decade and several articles have demonstrated an impact of IAP on the gut microbiota development. Several common findings highlighted the increment of Enterobacteriaceae and the reduction of strict anaerobes such as *Bifidobacterium* and *Bacteroides* genus ^128^. In addition, it has been observed a negative influence of IAP on the correct development of bifidobacterial community in newborns during the first months of life. Data accumulated during the last years suggest that IAP may also have lasting and significant effects on the infant’s resistome and mobilome. Two seminal studies demonstrated the impact of IAP on the prevalence of ARGs and mobile elements in vaginally-delivered full-term babies, showing also a beneficial effect of breastfeeding on the ARGs burden^15,129^. Most of the current studies concluded that the selective pressure exerted by IAP favors the presence of different bacteria carrying ARGs, most of them species belonging to Pseudomonadota phyla or *Staphylococcus* genus^128^. In addition, IAP has also been associated with increments of ARGs in other body microbiomes, such as the nasopharynx and oral microbiota^128^.

A recent study suggests that the use of broad-spectrum antibiotics for treating suspected early-onset neonatal sepsis (sEONS) has a significant impact on the microbial diversity, community composition, and selection of ARGs. The study found that the use of antibiotics resulted in a decrease in the abundance of *Bifidobacterium* species and an increase in the abundance of *Klebsiella* and *Enterococcus* species in the treated infants. The study also found that treatment with amoxicillin and cefotaxime had the strongest effect on both the microbial community composition and the ARG profile, while treatment with penicillin and gentamicin had the least effect^130^. In addition, *B. longum* and *B. breve* decreased in populations exposed to antibiotics, while *Bacteroides* increased^131^. Additionally, infants can inherit residual effects of past antibiotic use of the mother. Patangia *et al*. found that even before being exposed to antibiotics, newborns carry ARMs acquired from their mothers and the environment^71^. However, further research is required in this area.

### Effects of other resistomes: One-Health perspective

In order to understand ARG transmission, the development of the human gut resistome in early life and the crossover between mother and offspring resistome, it is necessary to refer to the One-Health perspective (Human-Animal-Environment)^132^. There is increasing concern that the use of antibiotics in food-producing and pet animals may be a source of antimicrobial resistance to humans^133^. In this sense, animal and environmental resistome may have a direct effect on the establishment and development of the human resistome. Indeed, having pets or animals at home has been found to influence the growth and development of gut bacteria^134,135^ and, therefore, this may affect the ARG carriage through the impact on the microbiota and the potential transference of strains harboring ARGs. The transfer of pathogens or antibiotic resistance genes from pets to humans has been documented^136–138^. Although antibiotic usage in animals is more and more regulated, antibiotics are still given to animals to treat disease and to not only prevent infection but also the propagation of disease to other animals^139^. In addition, there is evidence of environmental transmission of ARGs among livestock and humans. Similar to humans, antibiotics in animals cause changes in gut bacterial composition: a decrease in the species of *Lactobacillus*, *Bifidobacterium*, and *Ruminococcus* and a rise in *E. coli* levels were observed, leading to alterations in ARG levels^140^. In addition, the animal resistome is not only developed in antibiotic-treated animals; wild animals also have ARGs^141^.

Humans can be exposed to ARGs from animals by direct contact with them but also by consuming vegetables that have been cultivated on soil fertilized with manure and swine farm wastewater, or by consuming animal meat. Both manure and wastewater from livestock farms have been observed to contain residues of antibiotics and resistant microorganisms, such as *Proteobacteria, Firmicutes, Actinobacteria* and *Bacteroidetes* ^142^. Downstream, ARGs and antibiotic residues have been detected in vegetables grown in manure-amended soils, which then may reach human microbiome promoting horizontal gene transfer^143^. Animal meat is also a source of antibiotic resistance in humans. Even though there are strict biosecurity measures established, meat may still contain traces of antibiotics if the proper time for withdrawal antibiotics from the treated animals is not efficient. Moreover, the process of dressing a carcass may result in contamination with ARMs, which can come from sources such as the animal’s skin and gut contents, the hands of the workers, and the slaughter environment^141^. Therefore, it is important to consider that all ecological niches harboring microbial communities will contain ARGs, including aquatic environments and terrestrial environments (agricultural sites, wastewater plants) and also hospital environments such as the NCIU^144,145^. Therefore, the One-Health initiative bas to be brought to the forefront of ARG research.

## 5. How can we reduce antibiotic resistance in mother-infant dyads?

Apart from strategies that involve antibiotic stewardship^146^, recent studies have focused on developing new approaches to ameliorate the antibiotic resistance load in the human microbiome, but further research into new mechanisms to reduce the resistance load, especially in infants, are needed.

### Diet

Diet is significantly linked with the composition of microbiota, as it not only nourishes it but can also alter it, and, presumably, influence the resistome. Several studies have pointed the influence of maternal diet on their offspring gut microbiome, elucidating the role of the diet on the transfer of antibiotic resistances to the infant during delivery or lactation^147–149^. Recently, studies have started to explore the impact of diet on the dissemination of ARGs in the intestinal microbiota. For instance, it has been reported that a diverse, fiber-rich diet induces lower abundance of ARGs in human gut^150^. A diet high in sugar, fat, and protein can alter bacterial composition and diversity, increase the permeability of bacterial membranes, and activate the bacterial SOS response, resulting in gut inflammation and changes to gut bacteria and its by-products. These changes result in the amplification and transfer of exogenous ARGs in the gut^151^.

It has been established that different eating patterns, such as omnivore, ovolactovegetarian, and strict vegetarian diets, can have an impact on the resistome. It is believed that consuming fewer processed foods can help reduce the amount of ARGs in the gut microbiome^152^. However, other factors might also contribute to the resistome, as a study found that long-term dietary habits did not significantly affect resistome composition in the Dutch population^153^.

Therefore, diet seems to be a potential key factor on both acquisition and transmission of antibiotic resistances in all stages of life. For this reason, more knowledge is needed to develop dietary strategies that would help to ameliorate antibiotic resistances in the mother-infant environment.

### Dietary supplements: - biotics family

It’s becoming more than obvious that - biotics family including prebiotics and probiotics might have an important role in the restoration of gut dysbiosis and resilience. In this context, the utilization of probiotics appears to be a highly hopeful strategy in this field, either through simultaneous use with antibiotics to prevent disruption or by taking advantage of their benefits to heal the altered microbiome after antibiotics have caused harm. Probiotic administration to pregnant women is recommended by the World Allergy Organization (WAO) for pregnant women at high risk for having an allergic child and women who breastfeed infants at high risk of developing allergies. On the other hand, infants at high risk of developing allergies are also recommended to receive probiotic supplementation^154^. There is no evidence that taking probiotics or prebiotics during pregnancy causes infant and maternal adverse pregnancy outcomes^22^, but significantly modulates the maternal gut microbiota and immune status^23,24^.

The presence of *Bifidobacterium infantis* EVC001 in the infant gut microbiome leads to a decrease in the amount of ARGs and the types of bacteria that carry and potentially spread them among individuals^25^. In premature babies, probiotic supplementation may increase protection against harmful bacteria and reduce the negative impact of antibiotics on the microbiome and resistome of the gut^26^. A different study demonstrated that administering probiotics to preterm infants during hospitalization after birth decreased the gut microbiome’s resistance gene diversity and prevented its persistence^27^.

Another metagenomic analysis reported that taking a probiotic supplement consisting of commonly used species, reduced the amount of antibiotic resistance genes in the gut, however, this positive outcome was limited to only certain individuals who were receptive to probiotic colonization^28^.

The significance of considering the ecological environment in which probiotics are given is emphasized by these contrasting effects. On the other hand, prebiotics, which are not made from live bacteria, are unlikely to lead to an increase in resistance genes. As a result, prebiotics might be a valuable resource in the battle against antibiotic resistance and infection, although their effectiveness has yet to be confirmed^29^.

Further studies are required to assess the potential reduction of horizontal transfer of resistance genes by probiotic administration. In addition, more information about the use of prebiotics during pregnancy and lactation to prevent antibiotic resistance transfer to offspring is needed.

## 6. Conclusions and Future Perspectives

It is widely accepted that the early infant gut microbiota has a crucial role in infant health, and that the close relationship between mothers and their offspring determines the formation and development of the microbiota of the neonate. Therefore, several maternal and external factors may contribute to the modulation of the infant’s resistome. Mode of delivery, gestational age, antibiotic use, or type of lactation may facilitate the colonization of ARMs, leading to a high risk of infections in early life. The term resistome appears to be a concept that has spread worldwide as antibiotic resistance transmission rises to dangerous levels. As next generation sequencing techniques have improved in recent years, the scientific community has gained more knowledge on the mother-infant resistome. However, there are still some to questions to answer. Do babies acquire the first ARGs during birth or are they born with them? Is lactation a crucial factor in the acquisition of antibiotic resistance? What impact do the mother’s dietary and lifestyle habits have on her infant’s resistome? Which is the best approach to study the resistome? There are few studies attempting to answer these gaps in our knowledge, but there is still a lot of work to do. Delving into antibiotic resistance acquisition and transmission in early stages of life may allow us to develop new strategies to overcome and reduce the resistance load. Diet appears to be one of the main tools to control antibiotic resistance transmission, as the benefits of a healthy diet on human gut microbiota are well known. Prebiotics and probiotics may also help beneficial bacteria colonize infant guts, displacing ARMs, but more studies are needed to know if biotic intervention should be applied to pregnant women prophylactically and/or via newborn feeding. All approaches for the reduction of antibiotic resistance load in infants may start at the beginning of the pregnancy, or even before, so the resistome of pregnant women is important to be taken into account. This review aims to present the latest evidence on mother-infant antibiotic resistance and reveal the challenges that lay ahead in the fight against multidrug-resistant bacteria.

## Abbreviations

ARG: : antibiotic resistant genes
ARM: : antibiotic resistant microorganisms
HMO: : human milk oligosaccharides
MGE: : mobile genetic elements
WHO: : World Health Organization
CDC: : Centers for Disease Control and Prevention

## Data sharing

Data sharing is not applicable to this article, as no new data were created or analyzed in this report.
